# A case of neonatal-onset autoinflammatory syndrome with a *de novo PSMB9* mutation resembling Nakajo-Nishimura syndrome

**DOI:** 10.1186/1546-0096-13-S1-P183

**Published:** 2015-09-28

**Authors:** N Kinjo, N Kanazawa, H Mishima, A Kinoshita, K Yoshiura

**Affiliations:** 1 University of Ryukyus, Pediatrics, Nishihara-cho, Japan; 2 Wakayama Medical University, Dermatology, Wakayama, Japan; 3 Atomic Bomb Disease Institute, Nagasaki University, Human Genetics, Nagasaki, Japan

## 

We report a case of neonatal-onset autoinflammatory syndrome that resembles Nakajo-Nishimura syndrome (NNS), a rare autoinflammatory syndrome caused by a homozygous *PSMB8* mutation. The patient is a 7-year-old boy who demonstrated clinical findings that differ from those of typical NNS.

Exudative erythemas on his face, trunk, and extremities developed 2 weeks after birth. At 1 month of age, the patient developed fever, elevated serum C-reactive protein levels, and elevated hepatic amino transferase levels. A febrile convulsion caused basal ganglia calcification at 4 months of age. Severe pulmonary arterial hypertension of unknown origin (NT-proBNP 33,445 pg/mL) was diagnosed at 7 months of age. These symptoms improved after administration of bozentan and ambrisentan, which are endothelin receptor antagonists. At 9 months of age, he developed heliotrope rash, edematous erythema on his extremities, and myositis with elevated serum creatine kinase levels (16,000 IU/L). Antinuclear antibodies, as well as anti-Jo-1 and anti-DNA antibodies, were negative. Skin and muscle biopsies revealed massive cellular inflammation, consisting primarily of lymphocytes and monocytic cells, located in the epidermis and between muscle fibers with fat tissue necrosis in the subcutis. Concomitant degeneration and regeneration of muscle fibers occurred and resulted in moderate variations in the fiber size.

These results initially led to a diagnosis of juvenile dermatomyositis. His symptoms temporarily improved with corticosteroid treatment but recurred periodically. Administration of systemic agents such as azathioprine, methotrexate, cyclosporine, cyclophosphamide, mycophenolate mofetil, and intravenous immunoglobulins did not lead to remission.

Hepatosplenomegaly and liver cirrhosis due to pulmonary arterial hypertension induced portal hypertension beginning at age 6. The serum levels of interleukin (IL)-1β (28 pg/mL), IL-6 (84.3 pg/mL), TNFα (21.6 pg/mL), IL-18 (595 pg/mL), and IFN-γ (7.0 IU/mL) were all increased during the periodic recurrences. These periodic symptoms were refractory to several immunosuppressive agents, which suggested an autoinflammatory syndrome. Although presence of fever, erythemas, basal ganglia calcification and hepatosplenomegaly suggested NNS, some of the characteristic symptoms of NNS, such as lipomuscular atrophy in the upper body and long clubbed fingers with joint contractures, did not occur in this patient.

Since no significant mutations had been detected in the *NLRP3, NOD2, MEFV, MVK, TNFRSF1A*, and *PSMB8* genes, whole exome sequencing of the patient and his parents was performed and a novel *de novo* heterozygous mutation has been identified in *PSMB9,* encoding the immunoproteasome subunit beta1i, in the patient. Our case might represent a novel proteasome-associated autoinflammatory syndrome similar to but distinct from NNS, in which a heterozygous mutation of the *PSMB9* gene is responsible.

Written informated consent for publication of their clinical details was obtained from the patient/parent/guardian/relative of the patient.

